# Was hydrogen peroxide present before the arrival of oxygenic photosynthesis? The important role of iron(II) in the Archean ocean

**DOI:** 10.1016/j.redox.2023.103012

**Published:** 2023-12-25

**Authors:** Willem H. Koppenol, Helmut Sies

**Affiliations:** a Department of Chemistry and Applied Biosciences, Swiss Federal Institute of Technology, Zürich, Switzerland; b Institute for Biochemistry and Molecular Biology I, Medical Faculty, Heinrich-Heine-University Düsseldorf, Düsseldorf, Germany; c Leibniz Research Institute for Environmental Medicine, Düsseldorf, Germany

**Keywords:** Archean, Hydrogen peroxide, Radiation, oxidation‐reduction (redox), Fenton reaction

## Abstract

We address the chemical/biological history of H_2_O_2_ back at the times of the Archean eon (2.5–3.9 billion years ago (Gya)). During the Archean eon the *p*O_2_ was million-fold lower than the present *p*O_2_, starting to increase gradually from 2.3 until 0.6 Gya, when it reached ca. 0.2 bar. The observation that some anaerobic organisms can defend themselves against O_2_ has led to the view that early organisms could do the same before oxygenic photosynthesis had developed at about 3 Gya. This would require the anaerobic generation of H_2_O_2_, and here we examine the various mechanisms which were suggested in the literature for this. Given the concentration of Fe^2+^ at 20–200 μM in the Archean ocean, the estimated half-life of H_2_O_2_ is ca. 0.7 s. The oceanic H_2_O_2_ concentration was practically zero. We conclude that early organisms were not exposed to H_2_O_2_ before the arrival of oxygenic photosynthesis.

## 1 Introduction

Louis Jacques Thénard discovered H_2_O_2_ in 1818 while dissolving barium peroxide (BaO_2_) with acids [1], naming it *eau oxygénée*, oxygenated water. Initially seen solely as a chemical oxidant, H_2_O_2_ was, surprisingly, much later proven to play a significant role in biology and physiology as a messenger molecule.

Life depends on redox reactions, but these reactions are also essential for its regulation. Redox sensing and redox signaling reactions constitute what is known as redox regulation. A large body of knowledge has accumulated in recent years on this burgeoning topic [2,3]. Among the various biologically important redox metabolites, H_2_O_2_ plays a prominent role in this process [[4], [5], [6], [7]]. An exciting new insight of recent time has been that H_2_O_2_ is essential for performing basic physiological processes, covering aspects from fertilization to differentiation, proliferation, regeneration, repair, and normal metabolic functions. H_2_O_2_ is a major intracellular second messenger that mediates stress-inducible hormesis and programmed cell death [8]. This is substantially different from visualizing H_2_O_2_ solely as a damaging agent which, of course, it can be at an inappropriate concentration and/or location. At this point, it is appropriate to explain how an oxidizing molecule like H_2_O_2_ can play a role as messenger. It is a strong two-electron oxidant, but a weak one-electron oxidant. The two-electron electrode potential of +1.35 V at pH 7 [9] only comes into play if H_2_O_2_ is reduced at the surface of an electrode, or when it comes into contact with a two-electron donor such as a thiol or a selenol – in which case an oxygen atom is transferred. That reaction is followed by the transfer of reducing equivalents to restore the thiol or selenol, which constitutes the signalling process. As a one-electron oxidizing agent, H_2_O_2_ is a weak oxidant, *E*°′(H_2_O_2_, H^+^/HO^•^, H_2_O) being only +0.39 V at pH 7 [9], which is insufficient to oxidize amino acids, lipids, or nucleotides. Thus, this low one-electron electrode potential and the relatively slow kinetics of the oxygen transfer enable H_2_O_2_ to act as a signalling agent. The occurrence of H_2_O_2_ in mammalian systems as a physiological metabolite was established in 1970 [10], and an active research field developed [11]. A second boost in H_2_O_2_ research was initiated by the development of genetically encoded fluorescent probes to monitor H_2_O_2_ intracellularly [12]. Recent advances in this field have been summarized [13,14]. Among the four principles of the “Redox Code” by which biological systems are organized, the Third Principle includes activation/deactivation cycles of H_2_O_2_ production in redox signaling and its spatiotemporal organization [15].

All of this likely applied to life forms from today to about 550 million years ago, the Phanerozoic eon, when the *p*O_2_ had reached levels comparable to those today. But what about life forms during the Proterozoic eon (0.55–2.5 billion years ago, Gya), or the Archean eon (2.5–3.9 Gya)? The question arises as to when and how H_2_O_2_ came into play. During the Archean eon, one-third of Earth's history, the atmospheric O_2_ concentration was about 10^−6^ times that of the current level, which is essentially an anoxic environment [16,17]. Except for photochemical and radiolytic generation, the source of H_2_O_2_ in Archean time must have been the oxidation of H_2_O instead of the reduction of O_2_.

McKay and Hartman [18] suggested that photochemically produced H_2_O_2_ allowed early organisms to develop defenses against oxygen toxicity before the Great Oxidation Event (GOE), around 2.3 Gya. In line with that suggestion is the hypothesis that the last universal common ancestor (LUCA) was “able to tolerate O_2_ and detoxify ROS in a primordial environment” [19] where “ROS” stands for “reactive oxygen species”, an umbrella term whose use is being discouraged and to be superseded by referral to the specific molecular oxidant [20,21]. That H_2_O_2_ was present on Earth before O_2_ was generated by photosynthesis appears accepted, for instance because millimolar [22] concentrations of H_2_O_2_ are thought to be involved in the replication of primordial RNA. Even below the surface of Mars, anaerobic formation of H_2_O_2_ has been suggested [23]. One may ask, (i) what mechanisms existed that generate H_2_O_2_ in an anaerobic environment, (ii) what would happen to H_2_O_2_ in an anaerobic environment, and (iii) how would primitive organisms defend themselves? To answer these questions, we first address the conditions during the Archean, before the development of oxygenic photosynthesis, about 2.5 Gya. We then examine the proposed mechanisms for anaerobic H_2_O_2_ formation, the reaction of Fe^2+^, present in submillimolar concentrations in the Archean Ocean, with H_2_O_2_ and how this reaction is influenced by HCO_3_^−^. We will show that it is quite unlikely that early organisms were exposed to H_2_O_2_.

## 2 Archean conditions

Different approaches led to similar conclusions about the conditions on Earth 3 Gya (see Table 1).

**Table 1:** Comparison of present-day conditions and those 3 Gya. The concentrations refer to ocean water.

Quantity	Archean (3 Gya)	Today	Comment
Solar luminosity, *L*_⊙_	0.8	1	*L*_⊙_ = 3.9 × 10^26^ Watt
*p*O_2_, hPa	0.2•10^−6^	0.21	1.013 hPa = 1 atm
*p*CO_2_, hPa	0.1–0.2	0.04	(same)
pH, ocean	7	8.0	
*T*, K	≈290	288	
[Fe^2+^], ocean	0.02–0.2 mM	1 nM	Today: in upper oxygenated layers
[CO_2_], ocean	3–6 mM	0.1 mM	
[HCO_3_^−^], ocean	4–7 mM	0.63 mM	
[CO_3_^2−^], ocean	5.0–10 μM	5.8 μM	

See text for references. Concentrations involving CO_2_ were calculated with the program Visual Minteq 3.1.

The solar luminosity was *ca*. 80 % [24] which we mention here because it affected the photochemical generation of H_2_O_2_. The partial pressure of O_2_ was smaller by a factor of 10^6^ [16,17]. The temperature was not significantly different from today [25], because the lower luminosity of the sun was compensated for by the higher partial CO_2_ pressure [26]. The pH of the ocean was close to 7 [25,27,28], one pH unit lower than today. The concentration of Fe^2+^ may have been in the range of 0.02 mM [29] to 0.2 mM [30]. The partial pressure of CO_2_ was probably between 0.1 and 0.2 bar [25,27]. Under the assumption that the ionic strength of the ocean was similar to that of today, ca. 0.7 M, the solubility of CO_2_ would be about 10 % less than in pure water [31], and [CO_2_] would have been 3–6 mM. Given p*K*_a_s of 6.0 and 9.6 for H_2_CO_3_ at that ionic strength [32], those partial pressures lead to concentrations of HCO_3_^−^ between 3 and 6 mM and that of CO_3_^2−^ between 5 and 10 μM.

## 3 Origin of H_2_O_2_ in anaerobic environments

The current hypotheses for anaerobic formation of H_2_O_2_ are: (i) ionizing irradiation of H_2_O by ^40^K and U, (ii) atmospheric generation, namely via UV-photochemistry of CO_2_ and H_2_O and via ionization of CO_2_ by lightning, (iii) reaction of pyrite, FeS_2_, with H_2_O, (iv) reaction of quartz radicals with water and (v) condensation of H_2_O. We will use available Gibbs energies of formation (Δ_f_*G*°) and reaction (Δ_rxn_*G*°) and standard electrode potentials (*E*°) to decide whether proposed reactions are feasible. Thermodynamic quantities are obtained from the literature [9,[33], [34], [35], [36]] with one exception. The Δ_f_*G*° values of Fe^2+^ and Fe^3+^ in the tables of the National Institute of Science and Technology (NIST) [33] were revised [37], which led to a value of −160 kJ mol^−1^ for Δ_f_*G*°(FeS_2_), used here, rather than the NIST value of −167 kJ mol^−1^.

### 3.1 Ionizing irradiation

Radiolysis of H_2_O leads directly to H_2_O_2_:

(1) $$H_{2}O\;⟿\;H_{2}O*,H_{2}O^{•+},e^{‐}\;\to \;{\text{HO}}^{•},H^{•},e_{\text{aq}}^{‐},H^{+},H_{2},H_{2}O_{2}$$

The *G*-value of H_2_O_2_, or the number of molecules formed per 100 eV absorbed energy is 0.68. Said differently, ca. 0.73 μM H_2_O_2_ is formed per 10 Gray (Gy) or 1 krad [38]. Generation of H_2_O_2_ by ^40^K has been modelled with 87 reactions in ocean water of a composition thought to have been present 3.8 Gya [39]. This simulation was necessary because reactions other than direct formation influence the yield of H_2_O_2_. The result was a H_2_O_2_ concentration of ca. 25 μM after a period of 100 million years. A reaction of H_2_O_2_ with a Fe^2+^ was considered not important and was not included among these 87 reactions [39]. In contrast, Kasting et al. [26] earlier considered this reaction to be inevitable. Another simulation was made for a natural uranium reactor like the one in Oklo, Gabon, 2 Gya [40]. Due to a higher dose rate much more H_2_O_2_ is generated. These simulations contain illegal loops (D. Stanbury, personal communication) and can therefore be criticized for violating the principle of detailed balancing [41]. What is meant by that is that a forward reaction involves species that are not the same as those of the backward reaction, or that a reaction is present in a loop that is considered irreversible. We found no information on the generation of H_2_O_2_ by cosmic radiation. At the present time, it constitutes only a small fraction of all ionizing radiation received on Earth. Therefore, we assume that this process played only a minor role.

In conclusion, irradiation by ^40^K and U generates H_2_O_2_, but the exact amount is not known.

### 3.2 Atmospheric reactions

Atmospheric generation of H_2_O_2_ involves CO_2_ and H_2_O, lightning and UV irradiation [42]. The energetics refer to the gas phase.

(2) $${\text{CO}}_{2}+h\nu \;\to \;\text{CO}+O$$

(3) $$O+O\;\to \;O_{2}\;\Delta_{\text{rxn}}G{}^{\circ}=-463\;\text{kJ}/\text{mol}$$

(4) $$H_{2}O+h\nu \;\to \;{\text{HO}}^{•}+H^{•}$$

(5) H2O+O(D1)→2HO•ΔrxnG°=−124kJ/mol

(6) $$2{\text{HO}}^{•}+M\;\to \;H_{2}O_{2}+M\;\Delta_{\text{rxn}}G{}^{\circ}=-174\;\text{kJ}/\text{mol}$$

However, the following reaction sequence is considered to be more important [43]:

(7) $$H^{•}+O_{2}+M\;\to \;{\text{HO}}_{2}^{•}+M\;\Delta_{\text{rxn}}G{}^{\circ}=-182\;\text{kJ}/\text{mol}$$

(8) $$2\;{\text{HO}}_{2}^{•}\;\to \;H_{2}O_{2}+O_{2}\;\Delta_{\text{rxn}}G{}^{\circ}=-151\;\text{kJ}/\text{mol}$$

Pecoits et al. [43] modelled these and many other reactions and concluded that the rainout rate of H_2_O_2_ for a *p*CO_2_ of 0.1 bar was 2•10^5^ molecules cm^−2^s^−1^, and that rate was deemed insufficient for the oxidation and precipitation of the oldest Fe^3+^ formations more than 3 Gya. In contrast to Draganic [39], Pecoits et al. [43] carried out these simulations to find out how much Fe^2+^ would be oxidized by H_2_O_2_. They used a model that was developed by Kasting decades ago [44] that has been extended by him [45] and others [43,46] to include more atmospheric reactions. However, we could not find a recent description of this model. In the past, this model only had irreversible reactions, and we assume that the results are qualitatively correct, that is, H_2_O_2_ may have been formed in the atmosphere.

During the eon of interest, the Earth was not covered by ice, we thus do not discuss the possible storage of rained-out H_2_O_2_ in ice.

### 3.3 Generation of H_2_O_2_ by FeS_2_

Pyrite (FeS_2_) can and does generate H_2_O_2_ when O_2_ is present. The following Gibbs energy changes refer to pH 7.

(9) $${\text{FeS}}_{2}+\frac{15}{2}\;O_{2}+\frac{19}{2}\;H_{2}O\;\to \;\frac{1}{2}\;{\text{Fe}}_{2}O_{3}+2{\text{SO}}_{4}^{2-}+\frac{15}{2}\;H_{2}O_{2}+4H^{+}\;\Delta G{{}^{\circ}}^{\prime }=-620\;\text{kJ}\;{\text{mol}}^{-1}$$

Subsequently, pyrite reacts with H_2_O_2_:

(10) $${\text{FeS}}_{2}+\frac{15}{2}\;H_{2}O_{2}\;\to \;\frac{1}{2}\;{\text{Fe}}_{2}O_{3}+2{\text{SO}}_{4}^{2-}+\frac{11}{2}\;H_{2}O+4H^{+}\;\Delta G{{}^{\circ}}^{\prime }=-2166\;\text{kJ}\;{\text{mol}}^{-1}$$

But without O_2_ no H_2_O_2_ can be formed:

(11) $${\text{FeS}}_{2}+H_{2}O\;\to \;\text{FeS}+{\text{HO}}^{•}+S^{•-}+H^{+}\;\Delta G{{}^{\circ}}^{\prime }=+425\;\text{kJ}\;{\text{mol}}^{-1}$$

(12) $${\text{FeS}}_{2}+2\;H_{2}O\;\to \;\text{FeS}+H_{2}S+H_{2}O_{2}\;\Delta G{}^{\circ}=+374\;\text{kJ}\;{\text{mol}}^{-1}$$

**Fig. 1:**
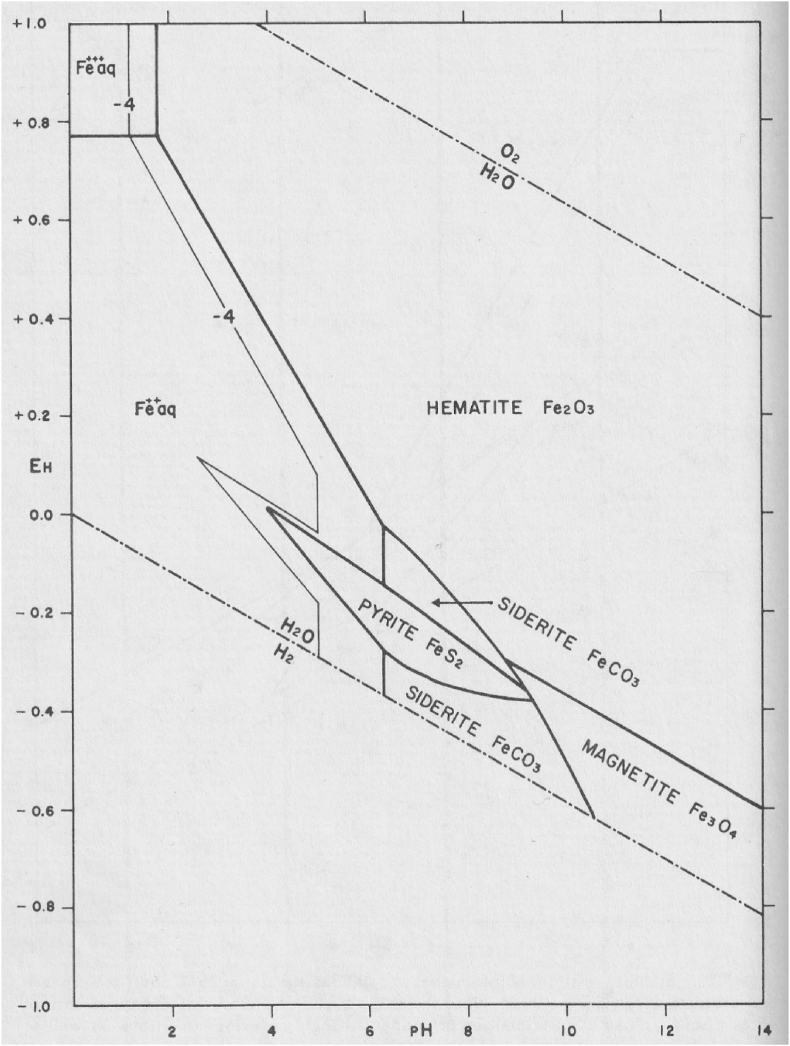
Pourbaix diagram of iron oxides, sulfides, and carbonate in water at 25 °C and 1 atm total pressure [49]. X-axis, pH; Y-axis, *E*°′. Total dissolved sulfur is 1 μM. Total dissolved carbonate is 1 M. Under Archean conditions, the carbonate concentration would be smaller and the fields of FeCO_3_ above and below FeS_2_ would be smaller. Concentration of Fe: 1•10^−6^ M (heavy line) and 1•10^−4^ M (thin line). The stability field of water is shown by the dash-dotted lines. The diagram shows that pyrite is stable in water from pH 4–9.5, at potentials less than 0.1 V and more than −0.35 V. The position of this stability field shows that pyrite is, by far, too weak to oxidize H_2_O to HO^•^, which requires +2.31 V at pH 7.

Nevertheless, anaerobic formation of H_2_O_2_ from pyrite was proposed by Schoonen and coworkers [47,48]. This proposal is surprising, given the location of pyrite in a Pourbaix diagram (Fig. 1) [49]: it is neither very oxidizing nor very reducing. If the location of pyrite in this diagram is correct, one expects pyrite to be formed in equilibrium with siderite (FeCO_3_), haematite (Fe_2_O_3_), and/or magnetite (Fe_3_O_4_). Such a microassembly of mineral phases has been documented. The haematite is thought to have been formed through oxidation of Fe^2+^ from a submarine hydrothermal vent by photosynthetically generated O_2_. The sample dates to 3.46 Gya [50].

According to the proposed mechanism [47,48], fracture of a FeS_2_ crystal breaks some of its S–S bonds which results in two S^•−^ radicals/Fe^2+^ on the surface. One of these radicals oxidizes a nearby Fe^2+^. The existence of Fe^3+^ on the surface of pyrite is not in doubt [51], and it is this cation that is thought to oxidize H_2_O to HO^•^. The implication is that the electrode potential of the Fe^3+^/Fe^2+^ couple at the surface is higher than *E*°′(HO^•^,H^+^/H_2_O), which is very high, +2.31 V at pH 7 [9]. One may ask why the sulfur radical S^•−^ did not oxidize H_2_O directly, as it must be even more oxidizing? One may further ask, why HO^•^ does not react with FeS_2_, but, according to the proposed mechanism, finds another HO^•^ to form H_2_O_2_ [47,52]? Thirdly, the proposed formation of H_2_O_2_ must be much faster than its documented [53] reaction with FeS_2_ such that a steady-state concentration is maintained that allows “detection”, which is also unlikely. The generation of H_2_O_2_ from H_2_O and FeS_2_ has been questioned before [54]. Indeed, the concentrations found vary widely. Javadi Nooshabadi et al. [55] reported H_2_O_2_ in the millimolar range under anaerobic conditions, but less under aerobic conditions. Zhang et al. [56] reported the opposite observation. Others [47,52,57] reported micromolar, not millimolar, concentrations of H_2_O_2_. It also has been reported [58] that anaerobic control experiments with pyrite did not produce significant HO^•^ within 60 min. The reports on the “anaerobic” formation of H_2_O_2_ should be seen in the light of the admission by Schoonen and coworkers in 2010 [59] that micromolar concentrations of O_2_ may have been present during their experiments. As shown above, FeS_2_ can thermodynamically reduce O_2_ to H_2_O_2_ and this has been experimentally observed [60].

Our conclusion is that, anaerobically, FeS_2_ does not generate H_2_O_2_.

### 3.4 Generation of H_2_O_2_ by minerals

Recently it has been suggested [61] that dangling quartz bonds may oxidize H_2_O to HO^•^. ESR evidence does show that there are radicals present in quartz. However, the method to determine H_2_O_2_ is severely flawed. He et al. [61] use a method published in 2005 [62], that endeavors to prevent a reaction between Fe^2+^ and H_2_O_2_ by chelation of Fe^2+^ with edta. However, three groups showed decades earlier that Fe^2+^edta reduces H_2_O_2_ with a rate constant of 7•10^3^ M^−1^s^−1^ [[63], [64], [65]]. Thus, edta likely enhanced the very reaction the authors [62] wanted to prevent. The analysis itself is based on the reaction of H_2_O_2_ with horseradish peroxidase, which then oxidizes leuco crystal violet; this process is followed spectrophotometrically. When this method did not work well at near pH 4–5, Schoonen and coworkers [62] speculated that edta did not chelate Fe^2+^ completely, which is incorrect [66]. In another publication [67], it was concluded that, anaerobically, high concentrations of H_2_O_2_ can be produced from Si^•^ or SiO^•^ radicals on the surface of crushed silicate rocks when water is added and heated to temperatures close to boiling point for 1 h or more. Below 80 °C little H_2_O_2_ is released. This is an extraordinary conclusion, given the decreased stability of H_2_O_2_ at higher temperatures [68] and the substantial presence of iron(II) in all samples examined: granite, basalt, and peridotite. H_2_O_2_ was determined by a well-established procedure [69], but the results should have been compared with those obtained by another method. Similar critique can be leveled at another report on the generation of H_2_O_2_ by quartz and silicate minerals [70]. The authors of the three papers questioned in this section as well as those who claimed that FeS_2_ produces H_2_O_2_ anaerobically should have spiked their samples with H_2_O_2_ to evaluate their analyses. All these authors claim that HO^•^ will react with another HO^•^ to form H_2_O_2_, which is only correct if there is nothing else in the solution that would react with HO^•^. Given that *E*°(HO^•^, H^+^/H_2_O) equals +2.31 V at pH 7 [9] and that it reacts rapidly with many compounds [71], significant formation of H_2_O_2_ is questionable.

While these authors have convincingly shown that radicals can be generated in minerals by breaking of bonds through mechanical stress, the hypothesis that dangling bonds generate H_2_O_2_ has not been proven.

### 3.5 Condensation of water

Recently, it was suggested that condensation of H_2_O(g) to droplets smaller than 10 μm generate H_2_O_2_ [72]. The authors report that a concentration of 68 μM H_2_O_2_ was detected. They did not address the energetics of condensation and H_2_O_2_ formation.

(13) $$H_{2}O\left(g\right)\;\to \;H_{2}O\left(l\right)\;\Delta_{\text{rxn}}G{}^{\circ}=-8.6\;\text{kJ}/\text{mol}$$

No reaction was proposed to account for the detected H_2_O_2_. We note that formation of H_2_O_2_ from water must also involve formation of H_2_, a reaction that is highly endergonic:

(14) $$2\;H_{2}O\;\to \;H_{2}O_{2}+H_{2}\;\Delta_{\text{rxn}}G{}^{\circ}=+340\;\text{kJ}/\text{mol}$$

H_2_ was not mentioned, and thermodynamic constraints were not addressed. The energy gained from condensation is insufficient − by far − to account for the formation of H_2_O_2_. Nevertheless, the authors write: “*Because H*_*2*_*O*_*2*_ *self-decomposes into H*_*2*_*O and O*_*2*_*, the results in this work suggest that water microdroplets, which are abundant in nature, might be an alternative source of atmospheric oxygen during the prebiotic era*” [72]**.** This report has been criticized by others, who argue that the detected H_2_O_2_ was generated by the procedure to generate water vapor, ultrasonic humidification [73].

Unless a convincing mechanism that includes formation of H_2_ has been formulated, we conclude that the condensation of water is unlikely to be a source of either H_2_O_2_ or O_2_.

### 3.6 Summary of anaerobic generation of H_2_O_2_

H_2_O_2_ may have been produced from ionizing radiation, UV-light and lightning.

## 4 What would happen to H_2_O_2_ in an anaerobic environment?

Given that the Archean ocean contained submillimolar concentrations of Fe^2+^, one would expect that HO^•^ is formed by the one-electron reduction of H_2_O_2_, the Fenton reaction:

(15) $${\text{Fe}}^{2+}+H_{2}O_{2}+H^{+}\;\to \;{\text{Fe}}^{3+}+{\text{HO}}^{•}+H_{2}O$$

**Scheme 1:**
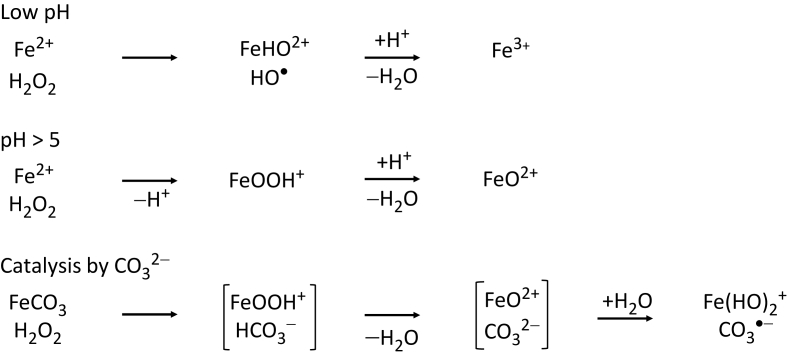
(Modified after [74]).

As written, reaction (15) is only valid below pH 5, with *k* = 53 M^−1^s^−1^. Above that pH, the rate of the Fenton reaction increases, because the reaction becomes first-order in [HO^−^]. This pH-dependence shows that the mechanism has changed. As reviewed [74], it is likely that above pH 5 the product of the Fenton reaction is oxidoiron(2+), FeO^2+^, also known by its non-systematic name ferryl. The Fenton reaction is also accelerated by HCO_3_^−^ above pH 5, and then yields CO_3_^•−^ [75], as reviewed (Scheme 1) [74]. CO_3_^•−^ is less oxidizing than HO^•^: while at pH 7, *E°*′(HO^•^/H_2_O) = +2.31 V [9], *E°*′(CO_3_^•−^/HCO_3_^−^) = +1.77 V [74]. The lower electrode potential may explain why CO_3_^•−^ reacts more slowly than HO^•^ with most organic molecules [71]. Another significant difference is that HO^•^ can add to, or oxidize, a molecule, while CO_3_^•−^ only oxidizes. The conclusion that CO_3_^•−^ is the product of the Fenton reaction under Archean conditions has ramifications: *in vivo* the *p*CO_2_ is similar to that during the Archean eon and therefore the Fenton reaction there also yields CO_3_^•−^.

The half-life of H_2_O_2_ in the Archean ocean can be estimated by the use of pseudo-first-order-kinetics. At pH 7, a rate constant of ∼10^4^ M^−1^s^−1^ in present day sea water has been measured for the Fenton reaction [76,77]. We consider this rate constant a minimum because [HCO_3_^−^] was larger in the Archean ocean (Table 1). For a Fe^2+^ concentration of approximately 0.1 mM [30], one obtains a half-life of 0.7 s. During this time, H_2_O_2_ can diffuse ca. 70 μm. This distance (root mean square) follows from RMS = (6*Dt*)^½^ with *D* = 1.1•10^−5^ cm^2^ s^−^^1^ at 20 °C [78,79]. After 5 half-lives virtually all H_2_O_2_ has reacted to form CO_3_^•−^. We now address the possible formation of another oxidant, HOOCO_2_^−^, (hydridodioxido)dioxidocarbonate(1−), reaction (16):

(16) $${\text{CO}}_{2}+H_{2}O_{2}\;⇄\;{\left[{\text{HCO}}_{4}\right]}^{-}+H^{+}$$

The kinetics and thermodynamics of this reaction [80] show that reaction (16) is uphill and slow. Furthermore, these data allow the calculation of the ratio HOOCO_2_^−^/H_2_O_2_ at pH 7 as 5.3•10^−3^ for a *p*CO_2_ of 0.1 atm. As the steady-state concentration of H_2_O_2_ would be vanishingly small, even more so would be that of HOOCO_2_^−^. It can therefore be dismissed as an oxidant in the Archean aqua sphere.

Any H_2_O_2_ is rapidly reduced by Fe^2+^ and yields CO_3_^•−^, which oxidizes another Fe^2+^. H_2_O_2_ would only survive in water without iron or other redox-active metal. However, this is unlikely, iron being the 4th most abundant element of the crust after O, Si, and Al. Thus, before oxygenic photosynthesis, organisms were not exposed to H_2_O_2_, HO^•^, O_2_^•−^, or CO_3_^•−^.

## 5 Oxidants and reductants were created by light

The first life forms, between approximately 4 and 3.5 Gya, had a metabolism based on H_2_. This could not have created oxidative stress, as *E*°′(H^+^/½H_2_) = −0.41 V. The origin of H_2_ is serpentinization, during which H^+^ is reduced by iron(2+) minerals [81]. H_2_ escaped from hydrothermal vents in the Archean ocean.

**Fig. 2:**
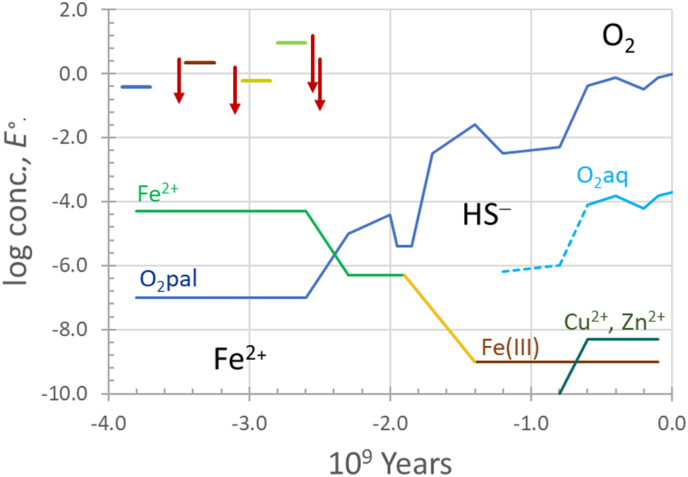
**Geochemical history of the Earth**. Initially, *p*O_2_ was exceptionally low (10^−7^ bar) and Fe^2+^ was present at a concentration of 0.02 mM–0.2 mM. This period is shown in the figure by **Fe**^**2+**^. The introduction of O_2_ by oxygenic photosynthesis may have started more than 3 Gya [82], some 700 million years before the GOE. These occurences are commonly referred to as “whiffs” of O_2_ [83]. The dating of such “whiffs” has recently become controversial. Obviously, the presence or absence of O_2_ at 3 Gya cannot be determined directly today. For that reason geochemists rely on proxies for O_2_, such as Mo. Mo(VI) is soluble and thus mobile. The 6+ oxidation state is thought to indicate the presence of O_2_. Upon reduction to Mo(IV) by H_2_S, necessarily in the absence of O_2_, one finds immobilized Mo(IV)–S species in sediments [84]. Diagenesis and metamorphism could invalidate this interpretation: if a “whiff” of oxygen occurred after sedimentation, and mobilized Mo, then the dating of the “whiff” is incorrect [85]. The GOE indicates the end of the Archean and the beginning of the Proterozoic eon. During the latter, Fe^2+^, present as Fe–S complexes, was oxidized to Fe_2_O_3_, and sulfidic sulfur to SO_4_^2−^. However, oxidation of Fe^2+^ and/or bacterial SO_4_^2−^ reduction likely led to an increase in HS^−^ [86], indicated as such in the figure. **O**_**2**_ indicates the end of the Proterozoic eon and extends to the present, during which *p*O_2_ is approximately at its present level, 0.2 bar. **Photosynthesis and concentrations**. Excitation of photosynthetic reaction centers causes these centers to become reducing, as indicated by downward arrows. Upper left, blue horizontal line: start of life based on H_2_ from serpentinization of at *E°*′(H^+^/1/2H_2_) = −0.41 V at pH 7. Red arrow at −3.5•10^9^ years: Fe-based photosynthesis (P870) and *E°*′ (Fe(HO)_2_^+^/Fe^2+^), +0.43 V, brown horizontal line. Red arrow at −3.1•10^9^ years: Sulfur-based photosynthesis (P840) and *E°*′(HS_2_^−^/2HS^−^), −0.23 V [36], yellow horizontal line. Red arrows at −2.5•10^9^ years, oxygenic photosynthesis by P680 and P700, and *E°*′(TyrO^•^/TyrOH), +0.96 V [87], green horizontal line. The electrode potentials depicted for the photosynthetic substrates Fe^2+^, H_2_S, and H_2_O reflect that electrons are transferred one, or possibly two, at a time. The electrode potentials of the photosynthetic reaction centers [88] (upper left) show that photosynthesis carried the risk of *oxidative* (P680) and *reductive* damage (P840). *p*O_2_ is depicted relative to the present atmospheric level, based on not only oxygenic photosynthesis, but also on a decline of O_2_ sinks that may involve magma degassing, mantle oxygen fugacity and the composition of the Earth's crust [89]. The increase in the *p*O_2_ over time is one of many found in the literature that all differ from each other with respect to the concentrations after the GOE, see for instances references [83,[90], [91], [92]]. Non-oxygenic photosynthesis with Fe^2+^ as substrate may have contributed to the massive Fe(III) formations around 3 Gya. The present-day nanomolar concentration of Fe^3+^ in sea water is possible only because of complexation by organic ligands. [Fe^3+^] would be lower, were it in equilibrium with haematite, Fe_2_O_3_. Cu^2+^ and Zn^2+^ were not available during the Proterozoic eon, being tied up as insoluble sulfides. O_2_aq is the calculated concentration in seawater based on O_2_pal (pal: present atmospheric level). Permanent oxygenation of the atmosphere occurred after 2.2 Gya [93]. (For interpretation of the references to colour in this figure legend, the reader is referred to the Web version of this article.)

About 3.3 Gya, there was the start of a rapid and extensive gene expansion, followed by gene loss, which peaked 3.1 Gya. This event represents a reprogramming of life forms [94]. It is around that time that non-oxygenic photosynthesis may have started [16] (see Fig. 2) under abyss(m)al light conditions near hydrothermal vents in the Archaic ocean [95]. It should be realized that light, and in a modulated form, photosynthesis has the potential to create strong oxidants and reductants. A photon with a wavelength of, say, 550 nm, green light, represents an energy (*U*) of 2.25 eV via *U* (eV) = 1240/λ (nm) which, in principle, is sufficient to break a chemical bond of 217 kJ mol^−1^. In that region of the spectrum, carotenes absorb light and transmit the energy of the photon through Förster resonance energy transfer to photosynthetic reaction centers that contain (bacterio)chlorophyll. Non-oxygenic photosynthesis, based on the oxidation of Fe^2+^ and H_2_S, occurred first and involves the reaction centers P840 (Reaction Centre 1) and P870 (Reaction Centre 2), respectively, so named because these bacteriochlorophylls absorb maximally at the wavelengths in nm listed after the P. We see that the energy of that photon with an energy of over 2 eV has been modulated to one below 1.5 eV. Carotenes also provide photoprotection [96,97], and act as antioxidants [98]. As such, they form the first line of defense against photooxidation. Upon excitation, the reaction center's bacteriochlorophyll becomes very reducing; it transfers an electron in a few picoseconds to either pheophytin, as in Reaction Centre 1, or to another bacteriochlorophyll, as in Reaction Centre 2. These pigments pass on the electron to other electron acceptors to generate NADH and/or ATP. The electrode potential of the Reaction Centre 1 P840^+^/P840 couple of green sulfur bacteria is ca. +0.25 V, while that of the Reaction Centre 2, *E*°(P870^+^/870) is slightly more oxidizing, +0.5 V [99]. Given these electrode potentials, oxidative damage is not expected.

The first photosynthetic process was likely based on the oxidation of Fe^2+^ to Fe(HO)_2_^+^, the metastable form of iron(3+) at pH 7 [20], and, as with H_2_, could not have led to oxidative or reductive stress, as *E°*′(Fe(HO)_2_^+^/Fe^2+^) = +0.35 V [89].

**Fig. 3:**
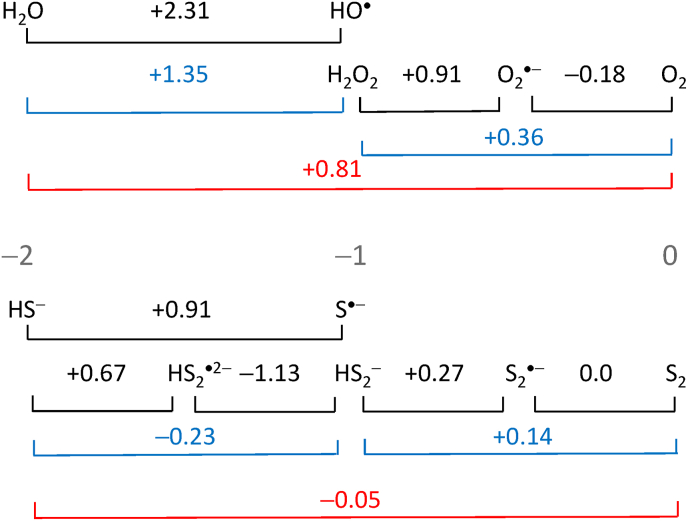
Latimer diagram of O and S. One-electron electrode potentials are in black, two-electron potentials in blue and four-electron potentials in red, all for pH 7, and relative to the Normal Hydrogen Electrode. The oxidation states are in grey. Data were taken from Refs. [9,36]. For most considerations, >2 electron electrode potentials are kinetically irrelevant, as electrons are transferred predominantly one at the time; two-electron events often involve transfer of O or S. (For interpretation of the references to colour in this figure legend, the reader is referred to the Web version of this article.)

Somewhat later, H_2_S was used as a reductant in photosynthesis (Fig. 2). The oxidation of H_2_S is a more complicated process, as the first one-electron oxidation is uphill (*E*°′ = +0.91 V (Fig. 3), while P840 only supplies +0.20 V (Fig. 2). It may be for that reason that the enzyme sulfide:quinone oxidoreductase catalyzes the oxidation in a single two-electron step [100]. For this reason the two-electron potential *E°*′(HS_2_^−^/2HS^−^), −0.23 V, rather than *E°*′(S^•−^/HS^−^), +0.91 V [36] is shown in Fig. 2 as a horizontal yellow line.

If one accepts the notion that protective enzymes were present in organisms before there was any O_2_ (see Introduction), then it would only be a small step to assume [101] that defenses against partially reduced analogs of S are necessary [102], because, as the reasoning goes, the chemical properties of such partially reduced sulfur species are like those of the corresponding oxygen species. To illustrate, catalase is considered necessary to catalyze the disproportionation of HS_2_^−^ to S_2_ and HS^−^. However, the redox chemistry of S could not be more different from O, as shown by the oxidation state vs *E*°′ diagrams of O [9] and S [36] at pH 7. These diagrams are summarized below (Fig. 3) as Latimer diagrams.

HS_2_^−^ is not oxidizing, furthermore, the reaction catalase supposedly catalyzes is not an exergonic disproportionation but an endergonic comproportionation [36]. Conceivably, this process would be driven by the subsequent reaction of S_2_ to S_n_.

Reductive damage might occur. The photosynthetic reaction center of green sulfur bacteria, P840, becomes very reducing upon excitation, −1.28 V [88], which could lead to reduction of disulfide bridges in proteins. The disulfanuidyl radical so formed, [RSSR]^•−^, is an oxidant (+0.80 V) and a reductant (−1.22 V) [36]; acquisition of an electron would result in breaking of disulfide bridge. One might ask whether such negative electrode potentials could lead to desulfurization, reaction (17). The answer is: probably not.

(17) $$\text{RSH}+H^{+}+e^{-}\;\to \;R^{•}+H_{2}S$$

The electrode potential of reaction (17) can be estimated as described before [103] with the Benson additivity method [104,105], and results in *E*°(RSH, H^+^/R^•^, H_2_S) = −1.1 V at pH 0 and *E°*′ = −1.5 V at pH 7, which is more negative than −1.22 V.

At the start of oxygenic photosynthesis and during early “whiffs” of O_2_ [16,83], O_2_ was reduced by Fe^2+^ and, ultimately, yielded CO_3_^•−^ as described above (Scheme 1). During these times, organisms had to learn to deal with O_2_ and its partially reduced forms. Oxygenic photosynthesis became firmly established 2.5 Gya [16]. *E*°(P680^+^/P680) = +1.2 V [88], which is considerable and allows the oxidation of tyrosine: *E*°′(TyrO^•^/TyrOH) = +0.97 V at pH 7 [87] (Fig. 2).

## 6 Defenses against O_2_

When O_2_ was introduced, being the “by-product” of oxygenic photosynthesis, organisms could not protect themselves against reactions that involve O_2_ itself or the partially reduced species H_2_O_2_ and O_2_^•−^. While currently obligate anaerobes may be able to do so, as mentioned in the Introduction, that ability may have been acquired through horizontal gene transfer. A benefit of oxygenic photosynthesis is that organisms could now use O_2_ as an electron acceptor to drive the endergonic synthesis of ATP; a process that would scavenge O_2_. O_2_, O_2_^•−^ and H_2_O_2_ are not very reactive: O_2_ reacts slowly because of its triplet ground state, O_2_^•−^ spontaneously and rapidly reacts with its protonated form, and H_2_O_2_ may have reacted rapidly with existing metalloenzymes. Singlet dioxygen (^1^Δ_g_) is reactive but decays to triplet dioxygen in a few μs. These are general statements and, as usual, the devil is in the detail. Ferredoxins, which are likely to have evolved during the Archean, when iron(II)-sulfide complexes may have been abundant, are vulnerable to these species. Damage to a protein can be repaired; damage to DNA may be fatal. Hypotheses on defenses against oxidative damage upon introduction of O_2_ during the Archean should start with a list of species that would have formed: O_2_^•−^, its disproportionation product H_2_O_2_, and CO_3_^•−^. HO^•^ does not belong in this list (Scheme 1) [74]. Re-reduction of Fe(III) species by O_2_^•−^ should not be mentioned: this reaction has never been experimentally observed with physiological ligands. The adjustment to growing or intermittent exposure to O_2_ by anaerobes has been reviewed [106,107]. Catalase and Fe-superoxide dismutase may have had different functions during the Archean than at present time. Cu/Zn superoxide dismutase likely emerged only late as Cu and Zn were not available during the sulfidic Proterozoic era (Fig. 2).

The introduction of oxygenic photosynthesis in evolution resulted in a paradox. Its “by-product”, O_2_, is toxic, but there is no better compound to drive the formation of ATP [108]. We conclude that a role for H_2_O_2_ as a messenger likely started after oxygenic photosynthesis became widespread.

## Funding

Not applicable.

## CRediT authorship contribution statement

**Willem H. Koppenol:** Writing – review & editing. **Helmut Sies:** Writing – review & editing.

## Declaration of competing interest

There is no conflict of interest.

## Data Availability

Data were obtained from the literature

## References

[1] Koppenol W.H., Sies H. (2018). Ancient molecule's 200th anniversary. Nature.

[2] Parvez S., Long M.J.C., Poganik J.R., Aye Y. (2018). Redox signaling by reactive electrophiles and oxidants. Chem. Rev..

[3] Sies H., Jones D.P. (2020). Reactive oxygen species (ROS) as pleiotropic physiological signalling agents. Nat. Rev. Mol. Cell Biol..

[4] Stone J.R., Yang S. (2006). Hydrogen peroxide: a signaling messenger. Antioxidants Redox Signal..

[5] Marinho H.S., Real C., Cyrne L., Soares H., Antunes F. (2014). Hydrogen peroxide sensing, signaling and regulation of transcription factors. Redox Biol..

[6] Sies H. (2017). Hydrogen peroxide as a central redox signaling molecule in physiological oxidative stress: oxidative eustress. Redox Biol..

[7] Di Marzo N., Chisci E., Giovannoni R. (2018). The role of hydrogen peroxide in redox-dependent signaling: homeostatic and pathological responses in mammalian cells. Cells.

[8] Zhou D.R., Eid R., Miller K.A., Boucher E., Mandato C.A., Greenwood M.T. (2019). Intracellular second messengers mediate stress inducible hormesis and Programmed Cell Death: a review. Biochim. Biophys. Acta Mol. Cell Res..

[9] Koppenol W.H., Stanbury D.M., Bounds P.L. (2010). Electrode potentials of partially reduced oxygen species, from dioxygen to water. Free Radical Biol. Med..

[10] Sies H., Chance B. (1970). The steady state level of catalase compound I in isolated hemoglobin-free perfused rat liver. FEBS Lett..

[11] Chance B., Sies H., Boveris A. (1979). Hydroperoxide metabolism in mammalian organs. Physiol. Rev..

[12] Belousov V.V., Fradkov A.F., Lukyanov K.A., Staroverov D.B., Shakhbazov K.S., Terskikh A.V., Lukyanov S. (2006). Genetically encoded fluorescent indicator for intracellular hydrogen peroxide. Nat. Methods.

[13] Stöcker S., Van Laer K., Mijuskovic A., Dick T.P. (2017). The conundrum of hydrogen peroxide signaling and the emerging role of peroxiredoxins as redox relay hubs. Antioxidants Redox Signal..

[14] Winterbourn C.C. (2018). Biological production, detection, and fate of hydrogen peroxide. Antioxidants Redox Signal..

[15] Jones D.P., Sies H. (2015). The redox code. Antioxidants Redox Signal..

[16] Lyons T.W., Reinhard C.T., Planavsky N.J. (2014). The rise of oxygen in Earth's early ocean and atmosphere. Nature.

[17] Catling D.C., Zahnle K.J. (2020). The Archean atmosphere. Sci. Adv..

[18] McKay C.P., Hartman H. (1991). Hydrogen peroxide and the evolution of oxygenic photosynthesis. Orig. Life Evol. Biosph..

[19] Slesak I., Slesak H., Kruk J. (2012). Oxygen and hydrogen peroxide in the early evolution of life on earth: *In silico* comparative analysis of biochemical pathways. Astrobiology.

[20] Koppenol W.H., Hider R.C. (2019). Iron and redox cycling. Do's and don'ts. Free Radical Biol. Med..

[21] Sies H., Belousov V.V., Chandel N.S., Davies M.J., Jones D.P., Mann G.E., Murphy M.P., Yamamoto M., Winterbourn C. (2022). Defining roles of specific reactive oxygen species (ROS) in cell biology and physiology. Nat. Rev. Mol. Cell Biol..

[22] Ball R., Brindley J. (2019). The power without the glory: multiple roles of hydrogen peroxide in mediating the origin of life. Astrobiology.

[23] Davila A.F., Fairén A.G., Gago-Duport L., Stoker C., Amils R., Bonaccorsi R., Zavaleta J., Lim D., Schulze-Makuch D., McKay C.P. (2008). Subsurface formation of oxidants on Mars and implications for the preservation of organic biosignatures. Earth Planet Sci. Lett..

[24] Gough D.O. (1981). Solar interior structure and luminosity variations. Sol. Phys..

[25] Krissansen-Totton J., Arney G.N., Catling D.C. (2018). Constraining the climate and ocean pH of the early Earth with a geological carbon cycle model. Proc. Natl. Acad. Sci. USA.

[26] Kasting J.F., Pollack J.B., Crisp D. (1984). Effects of high CO_2_ levels on surface temperature and atmospheric oxidation state of the early Earth. J. Atmos. Chem..

[27] Grotzinger J.P., Kasting J.F. (1993). New constraints on precambrian ocean composition. J. Geol..

[28] Halevy I., Bachan A. (2017). The geologic history of seawater pH. Science.

[29] Holland H.D. (1984).

[30] Walker J.C.G. (1983). Possible limits on the composition of the Archaean ocean. Nature.

[31] Scharlin P. (2012).

[32] Thurmond V., Millero F.J. (1982). Ionization of carbonic acid in sodium chloride solutions at 25-¦C. J. Solut. Chem..

[33] Wagman D.D., Evans W.H., Parker V.B., Schumm R.H., Halow I., Bailey S.M., Churney K.L., Nuttall R.L. (1982). Selected values for inorganic and C_1_ and C_2_ organic substances in SI units. J. Phys. Chem. Ref. Data.

[34] Lemire R.J., Berner U., Musikas C., Palmer D.A., Taylor P., Tochiyama O. (2013).

[35] Armstrong D.A., Huie R.E., Koppenol W.H., Lymar S.V., Merényi G., Neta P., Ruscic B., Stanbury D.M., Steenken S. (2015). Standard electrode potentials involving radicals in aqueous solution: inorganic radicals. Pure Appl. Chem..

[36] Koppenol W.H., Bounds P.L. (2017). Signaling by sulfur-containing molecules. Quantitative aspects. Arch. Biochem. Biophys..

[37] Rickard D., Luther G.W. (2007). Chemistry of iron sulfides. Chem. Rev..

[38] Wardman P. (2023). Initiating redox reactions by ionizing radiation: a versatile, selective and quantitative tool. Redox Biochem. Chem..

[39] Draganic I.G., Bjergbakke E., Draganic Z.D., Sehested K. (1991). Decomposition of ocean waters by potassium-40 radiation 3800 Ma ago as a source of oxygen and oxidizing species. Precambrian Res..

[40] Draganic I.G. (2005). Radiolysis of water: a look at its origin and occurrence in the nature. Radiat. Phys. Chem..

[41] Stanbury D.M. (2022). The principle of detailed balancing, the iron-catalyzed disproportionation of hydrogen peroxide, and the Fenton reaction. Dalton Trans..

[42] Haqq-Misra J., Kasting J.F., Lee S. (2011). Availability of O_2_ and H_2_O_2_ on pre-photosynthetic earth. Astrobiology.

[43] Pecoits E., Smith M.L., Catling D.C., Philippot P., Kappler A., Konhauser K.O. (2015). Atmospheric hydrogen peroxide and Eoarchean iron formations. Geobiology.

[44] Kasting J.F., Walker J.C.G. (1981). Limits on oxygen concentration in the prebiological atmosphere and the rate of abiotic fixation of nitrogen. J. Geophys. Res.: Oceans.

[45] Kasting J.F. (1982). Stability of ammonia in the primitive terrestrial atmosphere. J. Geophys. Res.: Oceans.

[46] Zahnle K., Claire M., Catling D. (2006). The loss of mass-independent fractionation in sulfur due to a Palaeoproterozoic collapse of atmospheric methane. Geobiology.

[47] Borda M.J., Elsetinow A.R., Schoonen M.A.A., Strongin D.R. (2001). Pyrite-induced hydrogen peroxide formation as a driving force in the evolution of photosynthetic organisms on an early Earth. Astrobiology.

[48] Borda M.J., Elsetinow A.R., Strongin D.R., Schoonen M.A. (2003). A mechanism for the production of hydroxyl radical at surface defect sites on pyrite. Geochim. Cosmochim. Acta.

[49] Garrels R.M., Christ C.L. (1965).

[50] Hoashi M., Bevacqua D.C., Otake T., Watanabe Y., Hickman A.H., Utsunomiya S., Ohmoto H. (2009). Primary haematite formation in an oxygenated sea 3.46 billion years ago. Nat. Geosci..

[51] Nesbitt H.W., Scaini M., Höchst H., Bancroft G.M., Schaufuss A.G., Szargan R. (2000). Synchrotron XPS evidence for Fe^2+^-S and Fe^3+^-S surface species on pyrite fracture-surfaces, and their 3D electronic states. Am. Mineral..

[52] Gil-Lozano C., Davila A.F., Losa-Adams E., Fairén A.G., Gago-Duport L. (2017). Quantifying Fenton reaction pathways driven by self-generated H_2_O_2_ on pyrite surfaces. Sci. Rep. - UK.

[53] McKibben M.A., Barnes H.L. (1986). Oxidation of pyrite in low temperature acidic solutions: rate laws and surface textures. Geochim. Cosmochim. Acta.

[54] Buckley A.N., Woods R. (2015). Can sulfide minerals oxidize water to hydrogen peroxide during grinding in the absence of dissolved oxygen?. Mining,Metall. Explor..

[55] Javadi Nooshabadi A., Larsson A.C., Kota H.R. (2013). Formation of hydrogen peroxide by pyrite and its influence on flotation. Minerals Eng.

[56] Zhang P., Yuan S., Liao P. (2016). Mechanisms of hydroxyl radical production from abiotic oxidation of pyrite under acidic conditions. Geochim. Cosmochim. Acta.

[57] Zhang P., Yuan S. (2017). Production of hydroxyl radicals from abiotic oxidation of pyrite by oxygen under circumneutral conditions in the presence of low-molecular-weight organic acids. Geochim. Cosmochim. Acta.

[58] Zhang P., Huang W., Ji Z., Zhou C., Yuan S. (2018). Mechanisms of hydroxyl radicals production from pyrite oxidation by hydrogen peroxide: surface versus aqueous reactions. Geochim. Cosmochim. Acta.

[59] Schoonen M.A.A., Harrington A.D., Laffers R., Strongin D.R. (2010). Role of hydrogen peroxide and hydroxyl radical in pyrite oxidation by molecular oxygen. Geochim. Cosmochim. Acta.

[60] Ahlberg E., Elfström Broo A. (1996). Oxygen reduction at sulphide minerals. 1. A rotating ring disc electrode (RRDE) study at galena and pyrite. Int. J. Miner. Process..

[61] He H., Wu X., Xian H., Zhu J., Yang Y., Lv Y., Li Y., Konhauser K.O. (2021). An abiotic source of Archean hydrogen peroxide and oxygen that pre-dates oxygenic photosynthesis. Nat. Commun..

[62] Cohn C.A., Pak A., Strongin D., Schoonen M.A. (2005). Quantifying hydrogen peroxide in iron-containing solutions using leuco crystal violet. Geochem. Trans..

[63] Sutton H.C., Winterbourn C.C. (1984). Chelated iron-catalyzed OH^.^ formation from paraquat radicals and H_2_O_2_: mechanism of formate oxidation. Arch. Biochem. Biophys..

[64] Rush J.D., Koppenol W.H. (1987). The reaction between ferrous aminopolycarboxylate complexes and hydrogen peroxide. An investigation of the reaction intermediates by stopped-flow spectrophotometry. J. Inorg. Biochem..

[65] Croft S., Gilbert B.C., Lindsay Smith J.R., Whitwood A.C. (1992). An E.S.R. investigation of the reactive intermediate generated in the reaction between Fe(II) and hydrogen peroxide in aqueous solution. Direct evidence for the formation of the hydroxyl radical. Free Radic. Res. Commun..

[66] Schwarzenbach G., Heller J. (1951). Komplexone XVIII. Die Eisen(II)- und Eisen(III)-komplexe der Äthylendiamin-tetraessigsäure und ihr Redoxgleichgewicht. Helv. Chim. Acta.

[67] Stone J., Edgar J.O., Gould J.A., Telling J. (2022). Tectonically-driven oxidant production in the hot biosphere. Nat. Commun..

[68] Schumb W.C., Satterfield C.N., Wentworth R.L. (1955). ACS Monograph Series.

[69] Baga A.N., Johnson G.R.A., Nazhat N.B., Saadalla-Nazhat R.A. (1988). A simple spectrophotometric determination of hydrogen peroxide at low concentrations in aqueous solution. Anal. Chim. Acta.

[70] He H., Wu X., Zhu J., Lin M., Lv Y., Xian H., Yang Y., Lin X., Li S., Li Y., Teng H.H., Thiemens M.H. (2023). A mineral-based origin of Earth's initial hydrogen peroxide and molecular oxygen. Proc. Natl. Acad. Sci. USA.

[71] National Institute of, Science and Technology NIST Chemical Kinetics Database, Standard Reference Database. http://kinetics.nist.gov/kinetics/index.jsp.2009.

[72] Lee J.K., Han H.S., Chaikasetsin S., Marron D.P., Waymouth R.M., Prinz F.B., Zare R.N. (2020). Condensing water vapor to droplets generates hydrogen peroxide. Proc. Natl. Acad. Sci. USA.

[73] Musskopf N.H., Gallo A.J., Zhang P., Petry J., Mishra H. (2021). The air−water interface of water microdroplets formed by ultrasonication or condensation does not produce H_2_O_2_. J. Phys. Chem. Lett..

[74] Koppenol W.H. (2022). Ferryl for real. The Fenton reaction near neutral pH. Dalton Trans..

[75] Illés E., Mizrahi A., Marks V., Meyerstein D. (2019). Carbonate-radical-anions, and not hydroxyl radicals, are the products of the Fenton reaction in neutral solutions containing bicarbonate. Free Radical Biol. Med..

[76] Moffett J.W., Zika R.G. (1987). Reaction kinetics of hydrogen peroxide with copper and iron in seawater. Environ. Sci. Technol..

[77] Millero F.J., Sotolongo S. (1989). The oxidation of Fe(II) with H_2_O_2_ in seawater. Geochim. Cosmochim. Acta.

[78] Stern K.G. (1933). Über die Diffusion des Hydroperoxyds in verschiedenen Lösungsmitteln. Ber..

[79] Borggaard O.K. (1972). Polarographic determination of diffusion coefficients of hydrogen peroxide and iron chelates and rate constants of hydroxyl radical reactions. Acta Chem. Scand..

[80] Bakhmutova-Albert E.V., Yao H., Denevan D.E., Richardson D.E. (2010). Kinetics and mechanism of peroxymonocarbonate formation. Inorg. Chem..

[81] Sleep N.H., Bird D.K., Pope E.C. (2011). Serpentinite and the dawn of life. Phil. Trans. Biol. Sci..

[82] Fournier G.P., Moore K.R., Rangel L.T., Payette J.G., Momper L., Bosak T. (2021). The Archean origin of oxygenic photosynthesis and extant cyanobacterial lineages. Proc. R. Soc. Lond. Ser. B.

[83] Anbar A.D., Duan Y., Lyons T.W., Arnold G.L., Kendall B., Creaser R.A., Kaufman A.J., Gordon G.W., Scott C., Garvin J., Buick R. (2007). A whiff of oxygen before the Great oxidation event?. Science.

[84] Paul K.M., van Helmond N.A.G.M., Slomp C.P., Jokinen S.A., Virtasalo J.J., Filipson H.L., Jilbert T. (2023). Sedimentary molybdenum and uranium: improving proxies for deoxygenation in coastal depositional environments. Chem. Geol..

[85] Slotznick S.P., Johnson J.E., Rasmussen B., Raub T.D., Webb S.M., Zi J.-W., Kirschvink J.L., Fischer W.W. (2022). Reexamination of 2.5-Ga "whiff" of oxygen interval points to anoxic ocean before GOE. Sci. Adv..

[86] Canfield D.E. (2004). The early history of atmospheric oxygen: homage to Robert M. Garrels. Annu. Rev. Earth Planet Sci..

[87] Mahmoudi L., Kissner R., Nauser T., Koppenol W.H. (2016). Electrode potentials of L-tryptophan, L-tyrosine, 3-nitro-L-tyrosine, 2,3-difluoro-L-tyrosine, and 2,3,5-trifluoro-L-tyrosine. Biochemistry.

[88] Hohmann-Marriott M.F., Blankenship R.E. (2011). Evolution of photosynthesis. Annu. Rev. Plant Biol..

[89] Chen G., Cheng Q., Lyons T.W., Shen J., Agterberg F., Huang N., Zhao M. (2022). Reconstructing Earth's atmospheric oxygenation history using machine learning. Nat. Commun..

[90] Mills D.B., Boyle R.A., Daines S.J., Sperling E.A., Pisani D., Donoghue P.C.J., Lenton T.M. (2022). Eukaryogenesis and oxygen in Earth history. Nat. Ecol. Evol..

[91] Allen J.F., Thake B., Martin W.F. (2019). Nitrogenase Inhibition limited oxygenation of Earth's proterozoic atmosphere. Trends Plant Sci..

[92] Stolper D.A., Keller C.B. (2018). A record of deep-ocean dissolved O_2_ from the oxidation state of iron in submarine basalts. Nature.

[93] Poulton S.W., Bekker A., Cumming V.M., Zerkle A.L., Canfield D.E., Johnston D.T. (2021). A 200-million-year delay in permanent atmospheric oxygenation. Nature.

[94] David L.A., Alm E.J. (2011). Rapid evolutionary innovation during an Archaean genetic expansion. Nature.

[95] Martin W.F., Bryant D.A., Beatty J.T. (2018). A physiological perspective on the origin and evolution of photosynthesis. FEMS (Fed. Eur. Microbiol. Soc.) Microbiol. Rev..

[96] Truscott T.G. (1990). New trends in photobiology: the photophysics and photochemistry of the carotenoids. J. Photochem. Photobiol. B Biol..

[97] Telfer A. (2005). Too much light? How β-carotene protects the photosystem II reaction centre. Photochem. Photobiol. Sci..

[98] Stahl W., Sies H. (2005). Bioactivity and protective effects of natural carotenoids. Biochim. Biophys. Acta, Mol. Basis Dis..

[99] Blankenship R.E. (2010). Early evolution of photosynthesis. Plant Physiol..

[100] Brito J.A., Sousa F.L., Stelter M., Bandeiras T.M., Vonrhein C., Teixeira M., Pereira M.M., Archer M. (2009). Structural and functional insights into sulfide:quinone oxidoreductase. Biochemistry.

[101] Olson K.R., Gao Y., Deleon E.R., Arif M., Arif F., Arora N., Straub K.D. (2017). Catalase as a sulfide-sulfur oxido-reductase: an ancient (and modern?) regulator of reactive sulfur species (RSS). Redox Biol..

[102] Olson K.R. (2020). Reactive oxygen species or reactive sulfur species: why we should consider the latter. J. Exp. Biol..

[103] Koppenol W.H. (1990). Oxyradical reactions: from bond-dissociation energies to reduction potentials. FEBS Lett..

[104] Benson S.W. (1976).

[105] Holmes J.L., Aubry C. (2012). Group additivity values for estimating the enthalpy of formation of organic compounds: an update and reappraisal. 2. C, H, N, O, S, and halogens. J. Phys. Chem. A.

[106] Khademian M., Imlay J.A. (2021). How microbes evolved to tolerate oxygen. Trends Microbiol..

[107] Lu Z., Imlay J.A. (2021). When anaerobes encounter oxygen: mechanisms of oxygen toxicity, tolerance and defence. Nat. Rev. Microbiol..

[108] George P., King T.E., Mason H.S., Morrison M. (1965). Oxidases and Related Redox Systems.

